# Selective *YAP1* Transcriptional Response to Graded Retinoic Acid Exposure in NTERA-2 Cells

**DOI:** 10.3390/life16101668

**Published:** 2026-10-06

**Authors:** Mustafa Ertan Ay, Kenan Çevik, Özlem İzci Ay

**Affiliations:** Department of Medical Biology and Genetics, Faculty of Medicine, Mersin University, 33343 Mersin, Türkiye; cvkknn@gmail.com (K.Ç.); ozlemay@mersin.edu.tr (Ö.İ.A.)

**Keywords:** retinoic acid, NTERA-2, *YAP1*/*WWTR1*/*NPTN*, miR-148a-3p, Hippo pathway, RT-qPCR

## Abstract

**Background:** Retinoic acid (RA) regulates neural cell-state transitions, but its effects on Hippo-pathway effectors in NTERA-2 cells remain incompletely defined. **Methods:** NTERA-2 cultures were exposed for seven days to 5, 10, or 15 µM RA, with untreated and 0.1% DMSO controls (*n* = 3 parallel flasks per group from the same passage and starting suspension). *YAP1*, *WWTR1*, *NPTN*, and miR-148a-3p expressions were quantified by RT-qPCR. RA-versus-DMSO comparisons were evaluated on the −ΔCq scale with false-discovery-rate correction and sensitivity analyses. **Results:** Metabolic viability remained at 95–97% of untreated-control values. Relative to DMSO, *YAP1* expression was 9.73-fold and 8.14-fold higher at 5 and 10 µM RA, respectively (*q* = 0.0462 for both), although levels remained below untreated control and direct RA-versus-untreated comparisons were nonsignificant. No corrected differences were detected for *WWTR1* or *NPTN*. miR-148a-3p comparisons were nonsignificant and interpreted cautiously because miR-26b-5p showed condition-dependent variation. **Conclusions:** Seven-day RA exposure was associated with a selective *YAP1* transcriptional response relative to the DMSO background at 5–10 µM. The pattern is also compatible with partial attenuation of a vehicle-associated reduction rather than induction above baseline. These transcript-level data do not establish neuronal maturation or Hippo-pathway activation.

## 1. Introduction

All-trans retinoic acid (ATRA; hereafter RA) is a vitamin A-derived morphogen that regulates gene transcription primarily through retinoic acid receptor (RAR)–retinoid X receptor (RXR) heterodimers and has a central role in neural patterning and cell-fate specification [1]. Its biological outcome is strongly context dependent: receptor composition, developmental stage, exposure duration and interactions with other signalling pathways can shift the response towards lineage commitment, differentiation or maintenance of less mature cellular states [1,2,3]. Experimental studies illustrate this stage dependence. In human pluripotent stem-cell differentiation protocols, RA treatment of hPSC-derived neural crest cells or neural crest precursors accelerates acquisition of vagal identity and early enteric neural progenitor specification [4], while cell-autonomous RA-receptor signalling exerts distinct effects at different stages of enteric nervous-system development [5]. In a primitive-streak-like system, tightly regulated endogenous RA levels controlled the emergence of neuroectodermal fates [6], and selective RARβ/RARγ activation altered specialization during stem-cell differentiation [7]. An early transcriptional response to RA should therefore not be regarded as equivalent to terminal neuronal maturation.

The human embryonal carcinoma line NTERA-2 cl.D1 is a well-established model for investigating RA-responsive neural development. Andrews first demonstrated that RA induces differentiation of this pluripotent human embryonal carcinoma line [8]. Subsequent studies showed that extended RA treatment, followed by enrichment and maturation procedures, can generate post-mitotic, polarized neurons [9] with neuronal marker expression, electrical activity and regional neural characteristics [10]. NTERA-2 cells can also generate neurons under neural stem-cell conditions and display radial-glia-like characteristics, underscoring the developmental heterogeneity of the model [11]. These studies make clear that the mature NT2 neuronal phenotype is the product of a prolonged, multistage protocol rather than RA exposure alone [9,10,11]. Cultures examined after seven days of RA treatment are therefore more appropriately viewed as a short-term RA-exposure model in which lineage-related transcription may be changing before a mature neuronal phenotype is established.

The Hippo pathway provides a plausible interface between RA-responsive cell-state transitions and the physical state of the culture. Its transcriptional effectors, yes-associated protein 1 (YAP1) and transcriptional coactivator with PDZ-binding motif (TAZ), encoded by *YAP1* and *WWTR1*, respectively, integrate biochemical signals with cell density, adhesion, cytoskeletal tension and extracellular-matrix mechanics [12,13]. In mouse embryonic neural stem cells, YAP/TAZ overexpression increased SOX2-positive ventricular-zone localization and neurosphere formation through TEAD-dependent mechanisms, supporting a role in neural stem-cell maintenance [14]. YAP/TAZ activity has also been shown to regulate neural progenitor number [15], while YAP1 promotes activation of adult hippocampal neural stem cells [16]. Collectively, these findings associate YAP/TAZ with neural progenitor competence and activation, but they do not imply that the two paralogues must respond identically at the transcript level.

Direct evidence from human neural models further suggests that the relationship between RA and YAP is stage-specific. Hindley et al. found that YAP was enriched in proliferative and less mature neural populations and was negatively associated with neuronal maturity. In NTERA-2 cells, RNA-interference-mediated YAP depletion enhanced neuronal differentiation. In a separate SH-SY5Y model, RA treatment and experimentally increased YAP activity cooperated to expand an early neural crest-like population and enhance migration [17]. These experiments establish biologically meaningful crosstalk between RA and YAP, but also show that increased YAP activity may accompany an early progenitor or neural crest-like state rather than mature neuronal differentiation. Whether RA exposure produces a coordinated *YAP1/WWTR1* response in NTERA-2 cells, or preferentially affects one effector, remains insufficiently defined.

Neuroplastin and microRNAs provide complementary perspectives on this question. *NPTN* encodes neuroplastin, a synaptic cell-adhesion glycoprotein implicated in neuronal development and plasticity [18]. Neuroplastin also forms functional complexes with plasma-membrane Ca^2+^-ATPases and contributes to the rapid clearance and regulation of neuronal Ca^2+^ signals, processes closely connected to glutamatergic synaptic plasticity [19,20]. Desrivières et al. reported that *NPTN* expression increased by approximately 1.5-fold at day 7 of differentiation in a human neural progenitor model, coinciding with developed neurites and induction of cell-adhesion and synaptic-transmission genes. The same study linked a cis-regulatory variant near *NPTN* to altered brain expression, cortical thickness and cognitive measures in adolescents [21]. *NPTN* may therefore help indicate whether an early transcriptional response is accompanied by a broader neural-organization programme, although its behaviour is likely to depend on the differentiation model and stage.

MicroRNAs add a post-transcriptional layer to RA signalling. Reviews have documented reciprocal relationships between RA signalling, miRNA expression and miRNA-biogenesis pathways [22,23]. Individual miRNAs can also participate functionally in RA-induced neuronal phenotypes: in Neuro-2A cells, miR-124 inhibition reduced RA-associated neurite outgrowth, whereas miR-124 overexpression promoted neuronal differentiation [24]. Nevertheless, such findings are miRNA- and model-specific. In a systematic review focused on neoplastic cell lines, Lima et al. identified 31 studies spanning nine tumour types, with RA concentrations and exposure periods ranging from 10^−3^ to 10^2^ µM and from 24 h to 21 days, respectively. More than 300 miRNAs were reported as RA-responsive, but the affected miRNAs varied markedly among cellular models and treatment conditions [25]. miR-148a-3p has shown neuroprotective or neuroinflammation-modulating effects in experimental contexts related to amyloid-β toxicity and traumatic brain injury [26,27], but these studies did not examine RA-treated NTERA-2 cells. Its response in the present model therefore required direct measurement rather than extrapolation.

The present study examined *YAP1*, *WWTR1*, *NPTN* and miR-148a-3p expression after seven days of exposure to 5, 10 or 15 µM RA in NTERA-2 cultures. Each RA group was compared with a matched 0.1% DMSO vehicle control, and culture morphology and MTT-based metabolic viability were assessed in parallel. By combining design-defined RA-versus-vehicle comparisons with concentration-trend, sensitivity and cross-target analyses, we asked whether graded RA exposure elicited a coordinated molecular response or a target-selective pattern. We anticipated that Hippo-effector transcription would be responsive to RA-associated state changes, but did not assume that *YAP1* and *WWTR1*, or the mRNA and miRNA targets, would change in parallel. 

## 2. Materials and Methods

### 2.1. Cell Line, Culture Conditions, and Retinoic Acid Treatment

Human NTERA-2 cells (NTERA-2 cl.D1; ATCC^®^ CRL-1973™, Manassas, VA, USA) were maintained in Dulbecco’s Modified Eagle Medium (DMEM; ATCC, Manassas, VA, USA) supplemented with 10% fetal bovine serum (Capricorn Scientific, Ebsdorfergrund, Germany), 2 mM L-glutamine (Capricorn Scientific, Ebsdorfergrund, Germany), 100 U/mL penicillin–streptomycin (Capricorn Scientific, Ebsdorfergrund, Germany), and 1% amphotericin B (Biological Industries, Beit HaEmek, Israel). Cells were cultured at 37 °C in a humidified atmosphere containing 5% CO_2_ and were routinely monitored by light microscopy for attachment, morphology, confluence, and overt signs of microbial contamination. The cell line was obtained directly from ATCC with lot-specific quality-control documentation confirming negative mycoplasma and sterility test results. No additional in-house mycoplasma assay was performed after thawing and expansion.

Cells from the same passage and a common homogeneous cell suspension were seeded into separate 25-cm^2^ culture flasks at approximately 3.3 × 10^5^ viable cells per flask in a total culture volume of 4 mL. Cells were allowed to attach for approximately 18–24 h before treatment. Five experimental conditions were established: untreated control (*n* = 3 flasks), dimethyl sulfoxide (DMSO) vehicle control (*n* = 3 flasks), 5 µM retinoic acid (RA; *n* = 3 flasks), 10 µM RA (*n* = 3 flasks), and 15 µM RA (*n* = 3 flasks). The three flasks per condition were maintained and processed as separate parallel culture units and capture flask-to-flask variability within the experimental batch; however, because they originated from the same passage and common starting suspension, they do not constitute fully independent experiments initiated from separate passages, cell preparations, or experimental days. The seeding density followed the original experimental workflow and was lower than the density commonly recommended for routine NTERA-2 maintenance; this difference, and the resulting potential influence of cell density on *YAP1/WWTR1* biology, are acknowledged as limitations.

All-trans retinoic acid (RA; R2625, Sigma-Aldrich, Taufkirchen, Germany) was dissolved in sterile cell-culture-grade DMSO (0231, Amresco, Solon, OH, USA) to prepare a 20 mM concentrated stock solution. Briefly, 12.02 mg RA was dissolved in DMSO to a final volume of 2 mL. From this concentrated stock, separate 5, 10, and 15 mM RA application stocks were prepared in DMSO. The solutions were divided into single-use aliquots, protected from light throughout preparation and handling, and stored frozen until use. Repeated freeze–thaw cycles were avoided.

For each treatment, the existing culture medium was completely removed and replaced with freshly prepared treatment medium. To obtain a final culture volume of 4 mL, 4 µL of the corresponding RA application stock was added to 3.996 mL of complete culture medium. Accordingly, the addition of 4 µL of the 5, 10, or 15 mM RA stock produced final nominal RA concentrations of 5, 10, or 15 µM, respectively. The DMSO vehicle group received 4 µL of pure DMSO in 3.996 mL of complete culture medium, whereas the untreated control group received 4 mL of complete culture medium without RA or DMSO. Thus, the final DMSO concentration was standardized at 0.1% (*v*/*v*) in the vehicle and all RA-treated groups.

The first RA or vehicle exposure was initiated after the initial cell-attachment period and was designated treatment day 0. Complete medium replacement with freshly prepared medium containing the corresponding RA concentration or vehicle was subsequently performed on treatment days 2, 4, and 6. RA was therefore not added cumulatively to the existing conditioned medium. The untreated control cultures underwent medium replacement according to the same schedule using complete medium without RA or DMSO. All experimental groups were processed at approximately the same time on each treatment day to minimize handling-related variability.

Cultures were maintained for a total treatment period of 7 days and were monitored microscopically throughout the experiment. Cells were harvested on treatment day 7, approximately 24 h after the final RA or vehicle exposure. Total RNA was subsequently isolated separately from each flask-level replicate and used for cDNA synthesis and targeted RT-qPCR analysis of *YAP1*, *WWTR1/TAZ*, *NPTN*, and miR-148a-3p expression.

### 2.2. MTT Cell Viability Assay

To assess whether the applied RA/DMSO conditions were associated with overt changes in metabolic viability, an MTT assay was performed (3-(4,5-dimethylthiazol-2-yl)-2,5-diphenyltetrazolium bromide). Briefly, NTERA-2 cells were seeded into 96-well plates at approximately 1 × 10^4^ cells/well in 100 µL DMEM and allowed to adhere for 24 h at 37 °C in 5% CO_2_. MTT was prepared at 5 mg/mL (0.005 g in 1 mL PBS), and 10 µL MTT solution was added to each well, followed by incubation for 4 h at 37 °C. The supernatant was then removed and 100 µL DMSO was added to dissolve formazan crystals. After 15–30 min incubation at room temperature, absorbance was measured using a BioTek ELX800 microplate reader (BioTek Instruments, Inc., Winooski, VT, USA) at 570 nm with 630 nm as the reference wavelength. Each condition was assessed in three technical wells. Viability was expressed as a percentage of the untreated control. The assay was used as a descriptive measure of metabolic viability and was not designed as an independent proliferation-rate assay.

### 2.3. RNA Isolation, Quality Assessment, and cDNA Synthesis

Total RNA was isolated from NTERA-2 cells using TRIzol reagent (Invitrogen, Thermo Fisher Scientific, Waltham, MA, USA) according to the manufacturer’s instructions. Following isolation, RNA samples were treated with DNase to reduce potential genomic DNA contamination. RNA concentration and purity were assessed using a NanoDrop 2000 spectrophotometer (Thermo Fisher Scientific, Waltham, MA, USA), while gross RNA integrity and possible degradation were evaluated by denaturing agarose gel electrophoresis. Only RNA samples considered suitable based on these quality assessments were used for reverse transcription, with equal amounts of total RNA used across samples.

For the mRNA targets *YAP1*, *WWTR1*, and *NPTN*, cDNA was synthesized using random hexamer primers and RevertAid Reverse Transcriptase (Thermo Fisher Scientific, Vilnius, Lithuania) according to the manufacturer’s instructions. For miRNA analysis, mature miR-148a-3p and the endogenous reference miR-26b-5p were reverse-transcribed separately using sequence-specific reverse-transcription primers designed for the corresponding mature miRNA sequences. Reverse transcription was performed at 42 °C for 60 min, followed by enzyme inactivation at 70 °C for 5 min. The resulting cDNAs were stored at −20 °C until quantitative PCR analysis.

### 2.4. Primer and Probe Design and Validation

Gene-specific primers and TaqMan probes were designed using Primer Express software version 3.0 (Applied Biosystems, Foster City, CA, USA). For mRNA targets, oligonucleotides were preferentially positioned within regions shared by the relevant annotated transcript isoforms, where feasible, to provide broad coverage of the intended transcripts. Primer- and probe-binding regions were screened against known sequence variants available in NCBI databases at the time of assay design, and regions overlapping known single-nucleotide polymorphisms were avoided whenever possible. Candidate sequences were also evaluated for favourable predicted amplification characteristics and assessed using NCBI BLAST (https://blast.ncbi.nlm.nih.gov/Blast.cgi; accessed on 5 June 2026) to minimize nonspecific amplification. Only sequences demonstrating suitable design characteristics and high predicted target specificity were selected for synthesis.

Primers and probes, including the modified miRNA probes, were custom-synthesized by Sentebiolab Genetics (Ankara, Türkiye). For the miR-148a-3p and miR-26b-5p assays, selected cytosine residues within the probes were replaced with C-5-propynyl-deoxycytidine (pdC) to increase hybridization affinity and duplex stability. The pdC-modified probes were used in combination with TaqMan chemistry to enhance hybridization specificity.

Before analysis of the experimental samples, all primer–probe assays were evaluated using Total RNA Control (Human) (Applied Biosystems, Thermo Fisher Scientific, Foster City, CA, USA; Cat. No. 4307281). Only assays producing consistent and reproducible amplification with acceptable amplification-curve characteristics were retained; assays failing to meet these criteria were excluded. The primer and probe sequences used in the final RT-qPCR assays are presented in Supplementary Table S1.

### 2.5. Quantitative Real-Time PCR

The expression levels of *YAP1* (*Yes1 associated transcriptional regulator*), *WWTR1* (*WW domain-containing transcription regulator 1, encoding TAZ*), *NPTN* (*neuroplastin*), and miR-148a-3p were quantified by quantitative real-time PCR using an ABI Prism 7500 Real-Time PCR System (Applied Biosystems, Thermo Fisher Scientific, Foster City, CA, USA). *ACTB* was used as the endogenous reference for *YAP1*, *WWTR1*, and *NPTN*, whereas miR-26b-5p was used as the endogenous reference for miR-148a-3p. *ACTB* is among the most frequently used reference genes in vertebrate RT-qPCR studies, although its suitability remains experimental-context dependent [28]. miR-26b-5p has previously been evaluated among candidate endogenous controls for miRNA RT-qPCR [29]; however, it has also been implicated in neuronal differentiation [30]. Therefore, the available raw Cq distributions for both normalizers were retrospectively examined across all experimental groups to assess potential condition-dependent variation in reference expression (Supplementary Table S2).

Each PCR reaction was performed in a final volume of 25 µL containing 12.5 µL of TaqMan™ Universal PCR Master Mix (Applied Biosystems, Thermo Fisher Scientific, Foster City, CA, USA), 5 µL of cDNA, gene-specific forward and reverse primers at a final concentration of 455.3 nM each, a TaqMan probe at a final concentration of 342.9 nM, and nuclease-free water to the final volume. Thermal cycling consisted of an initial incubation at 50 °C for 2 min and enzyme activation at 95 °C for 10 min, followed by 50 cycles of denaturation at 95 °C for 15 s and annealing/extension at 60 °C for 1 min.

Each flask-level sample was analysed separately, and PCR reactions were performed in technical triplicate. Technical replicate Cq values were evaluated together with their corresponding amplification curves, and acceptable replicates were averaged for subsequent analysis. No-template controls were included in each PCR run and showed no detectable amplification. Reverse-transcriptase-negative controls were not included, which is acknowledged as a methodological limitation.

Relative expression was calculated using the comparative Cq (2^−ΔΔCq^) method, with *ACTB* or miR-26b-5p serving as the corresponding endogenous reference. An assay-appropriate pooled cDNA calibrator generated from the same pooled RNA source was used for relative quantification. Consequently, untreated-control values were not necessarily expected to be centered at 1 on the relative-quantity scale. Normalized −ΔCq values were used for inferential statistical analyses; between-group differences on the −ΔCq scale were expressed as log_2_ fold changes and converted to fold changes by exponentiation (2^(log^_2_^difference)^) for biological interpretation.

### 2.6. Statistical Analysis

Statistical analyses were performed using Python version 3.12.13 (Python Software Foundation, Wilmington, DE, USA), with NumPy 2.3.5, pandas 2.2.3, and SciPy 1.17.0. Figures were generated using Matplotlib 3.10.8 and seaborn 0.13.2. Gene and miRNA expression were analysed on the −ΔCq scale, with higher values indicating greater normalized expression. Overall differences among the five experimental groups were evaluated using Welch’s ANOVA, the Kruskal–Wallis test, and permutation-based analysis of variance. Welch-based procedures were used as the primary parametric analyses because they do not require equality of group variances, an important consideration with small groups and visibly heterogeneous dispersion; nonparametric and permutation analyses were retained as complementary robustness checks. The primary pairwise contrasts were defined by the experimental design as each RA concentration versus the common DMSO vehicle before inferential testing of those contrasts; the study was not preregistered. These contrasts were assessed using Welch’s *t*-test, supplemented by bootstrap 95% confidence intervals, Hedges’ *g*, Cliff’s delta (*δ*), exact permutation tests, and leave-one-out sensitivity analyses. *YAP1* at each RA concentration was additionally compared directly with the untreated control. Linear and quadratic concentration trends were assessed using regression models with heteroscedasticity-consistent HC3 standard errors. Cross-target relationships were examined using Spearman’s rank correlation, and the overall expression structure was explored using principal component analysis. Benjamini–Hochberg correction was applied separately to predefined analytical families: the four target-wise Welch omnibus tests; the four target-wise permutation omnibus tests; the 12 RA-versus-DMSO pairwise comparisons (four targets × three RA concentrations); the four DMSO-versus-untreated comparisons; the three secondary *YAP1* RA-versus-untreated comparisons; the four linear trends; the four quadratic trends; and the six cross-target correlations. All tests were two-sided, and an FDR-adjusted *q*-value < 0.05 was considered statistically significant. Raw *ACTB* and miR-26b-5p Cq distributions were assessed retrospectively across the five conditions using Welch’s ANOVA and the Kruskal–Wallis test; these analyses were considered a descriptive stability assessment rather than formal multi-reference-gene validation. MTT percentages were evaluated descriptively because only technical-well values, not independent experiment-level measurements, were available.

### 2.7. Use of Generative AI-Assisted Tools

Generative AI-assisted tools were used to support statistical code development, data presentation, English-language editing, and manuscript organization. All analytical procedures were executed using the software and packages specified above, and all derived results, tables, figures, and textual outputs were reviewed and verified by the authors against the source data. Generative AI was not used to produce or alter raw experimental data or to make autonomous scientific decisions.

## 3. Results

### 3.1. Culture Morphology and MTT-Based Metabolic Viability

**Figure 1:**
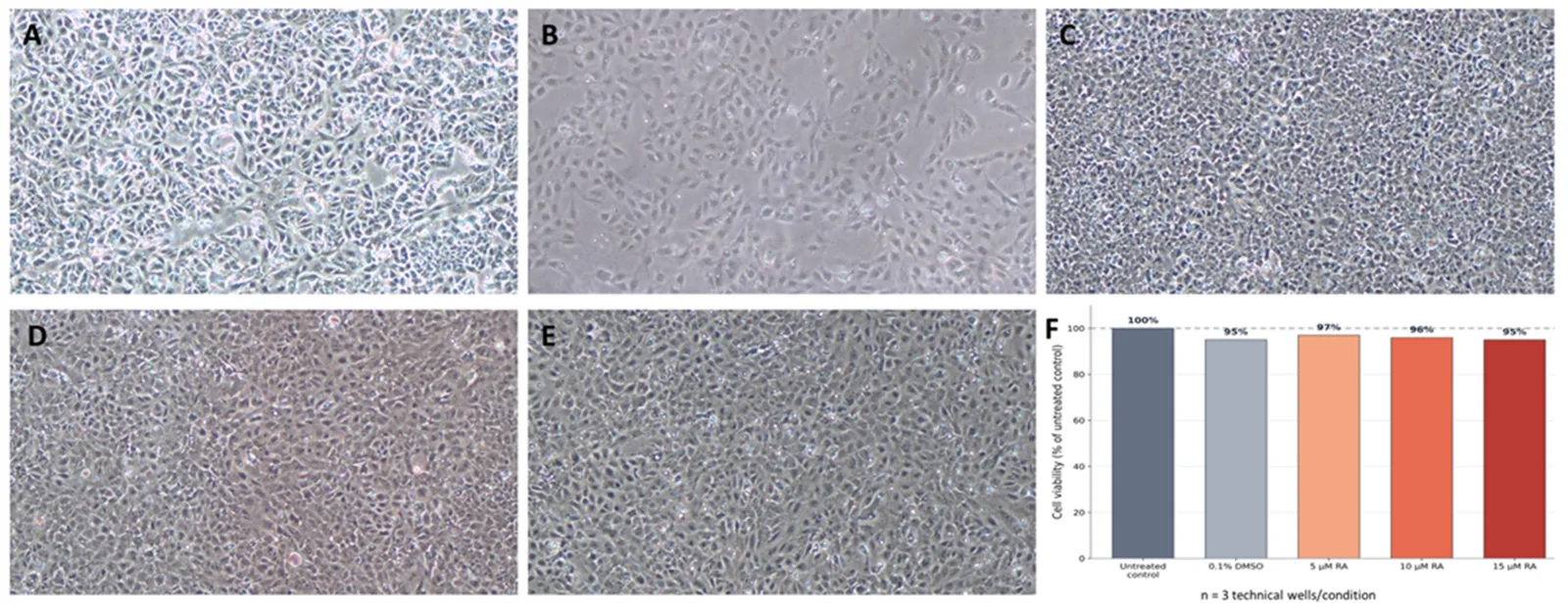
Representative morphology and metabolic viability of NTERA-2 cultures following retinoic acid exposure. Representative phase-contrast micrographs of NTERA-2 cells after seven days under the following conditions: (**A**) untreated control, (**B**) 0.1% DMSO vehicle control, (**C**) 5 µM RA, (**D**) 10 µM RA, and (**E**) 15 µM RA. All micrographs were acquired using an Olympus BX50 microscope (Olympus Corporation, Tokyo, Japan) with a 4× objective and are displayed at identical magnification. Scale bars are not shown because the image-calibration data required for retrospective spatial calibration were unavailable. (**F**) MTT-based metabolic viability normalized to the untreated control (100%). Relative viability was 95% for DMSO and 97%, 96%, and 95% for 5, 10, and 15 µM RA, respectively. The MTT assay was performed using three technical wells per condition (*n* = 3 technical wells/condition) and is presented descriptively without inferential statistical analysis. The micrographs are representative qualitative observations and were not subjected to quantitative morphometric analysis.

Representative phase-contrast images obtained after seven days of treatment showed predominantly adherent NTERA-2 cultures in the untreated control, vehicle control, and all RA-treated groups (Figure 1A–E). In the representative fields, the DMSO field showed cell spreading and intercellular spacing, while the untreated-control field had an intermediate appearance and the RA-treated fields appeared more densely populated and spatially compact. Some heterogeneity in cell spreading was also evident across conditions. These observations were qualitative only; confluence, cell number, cell area, neurite-related features, and proliferation were not quantified. Accordingly, the micrographs are presented as descriptive context rather than as evidence of a treatment-specific morphometric phenotype.

MTT-based metabolic viability remained close to the untreated-control reference across all experimental conditions (Figure 1F). Relative viability was 100% in the untreated control, 95% in the 0.1% DMSO vehicle group, and 97%, 96%, and 95% following exposure to 5, 10, and 15 µM RA, respectively. Thus, no marked reduction in metabolic viability was observed under the tested conditions. Measurements were obtained from three technical wells per condition within the assay; independent experiment-level measurements required for inferential analysis were unavailable. The MTT result was therefore evaluated descriptively and should not be interpreted as a direct measurement of proliferation rate.

### 3.2. Distribution and Overall Comparison of Normalized Expression

**Figure 2:**
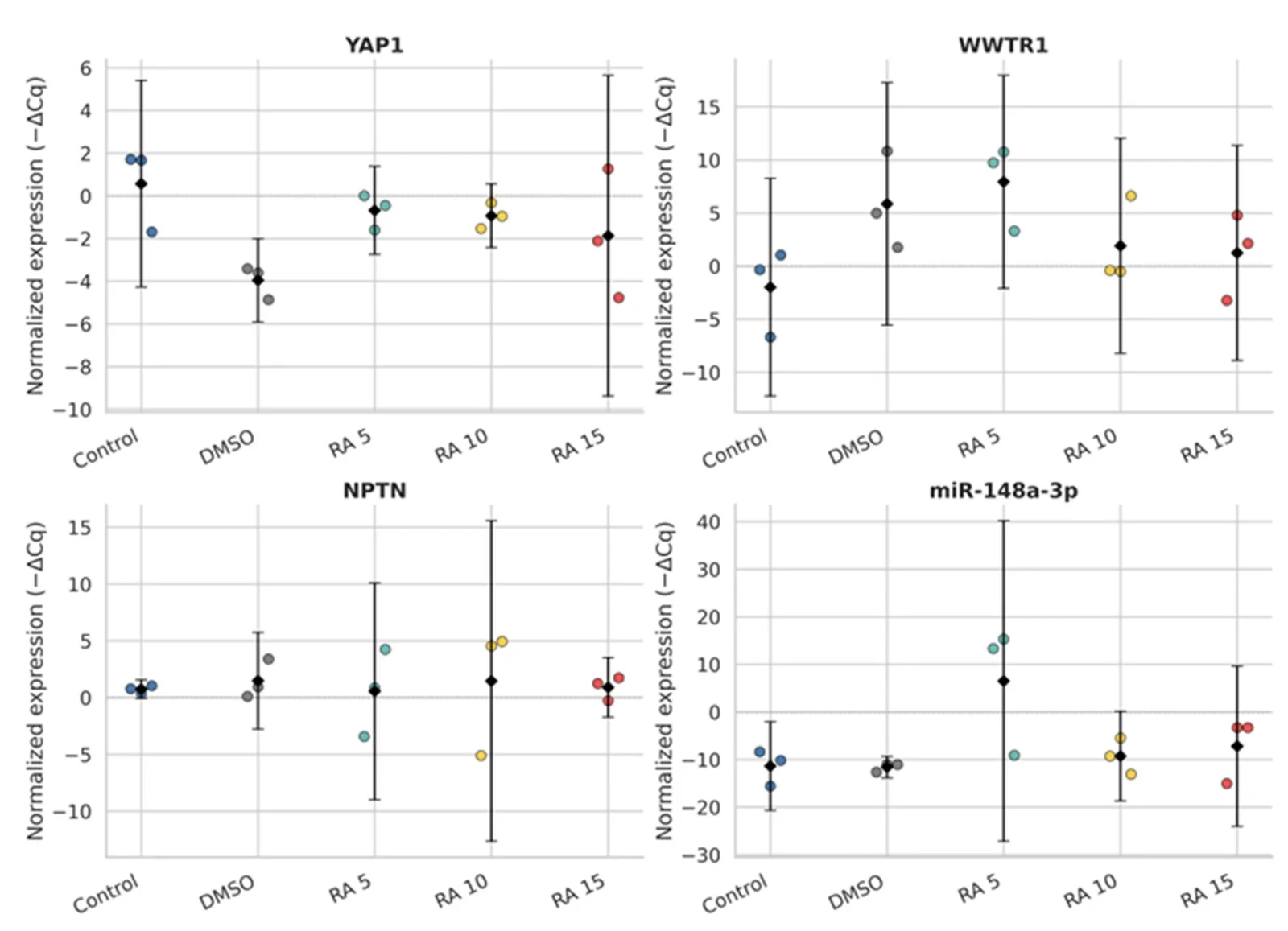
Normalized expression of *YAP1*, *WWTR1*, *NPTN*, and miR-148a-3p across experimental conditions. Expression is presented as −ΔCq values in the control, 0.1% DMSO, and 5, 10, and 15 µM RA groups. Coloured circles represent individual flask-level replicates (*n* = 3 per condition), black diamonds indicate group means, and error bars show 95% confidence intervals. Higher −ΔCq values indicate greater normalized expression.

Normalized expression was evaluated on the −ΔCq scale, with higher values indicating greater target expression relative to the corresponding endogenous normalizer. Each experimental condition comprised three parallel flask-level replicates derived from the same experimental batch. Descriptive statistics and DMSO-referenced fold changes are presented in Table 1, while the distributions of the individual flask-level measurements are shown in Figure 2.

The mean *YAP1* −ΔCq value was −3.960 ± 0.786 in the DMSO group and increased to −0.678 ± 0.831, −0.936 ± 0.600, and −1.870 ± 3.024 in the 5, 10, and 15 µM RA groups, respectively. These differences corresponded to DMSO-referenced fold changes of 9.73, 8.14, and 4.26. The dispersion was relatively limited at 5 and 10 µM RA but increased substantially at 15 µM RA (Table 1 and Figure 2).

*WWTR1* expression showed considerable flask-to-flask variability across all conditions. Relative to DMSO, the estimated fold changes were 4.19 at 5 µM RA, 0.065 at 10 µM RA, and 0.041 at 15 µM RA. However, the wide within-group distributions indicated that these estimates were not stable across the flask-level measurements. *NPTN* expression remained broadly comparable between the DMSO and RA-treated groups, with fold changes of 0.53, 0.99, and 0.67 at 5, 10, and 15 µM RA, respectively.

The most pronounced heterogeneity was observed for miR-148a-3p at 5 µM RA. Two cultures showed marked increases, whereas the third remained closer to the vehicle control range. Consequently, the mean-based fold-change estimate was extremely large but accompanied by substantial dispersion and should not be interpreted as a uniform induction across flask-level replicates (Table 1 and Figure 2).

In the five-group omnibus analyses, *YAP1* showed a nominal difference according to Welch’s test (*F* = 6.742, *p* = 0.032), but this result did not remain significant after BH-FDR correction (*q* = 0.130). The corresponding permutation test (*p* = 0.086) and Kruskal–Wallis test (*H* = 7.233, *p* = 0.124) were also not significant. No corrected omnibus effect was detected for *WWTR1*, *NPTN*, or miR-148a-3p (all *q* > 0.13; Table 2). Thus, none of the four targets showed a statistically robust overall difference across all five experimental groups after multiplicity correction.

### 3.3. Design-Defined Vehicle Comparisons and Secondary Untreated-Control Comparisons

The design-defined RA-versus-DMSO comparisons identified the clearest expression differences for *YAP1* (Table 3). At 5 µM RA, the mean *YAP1* difference was 3.282 log_2_ units, corresponding to a 9.73-fold higher level relative to DMSO (95% bootstrap CI for the −ΔCq difference: 2.263–4.301; Welch *p* = 0.0077; BH-adjusted *q* = 0.046). The standardized effect was large (Hedges’ *g* = 3.25).

Similarly, 10 µM RA produced a mean *YAP1* difference of 3.024 log_2_ units and an estimated 8.14-fold higher level relative to DMSO (95% bootstrap CI: 2.142–3.924; Welch *p* = 0.0073; *q* = 0.046; Hedges’ *g* = 3.46). In contrast, the estimated 4.26-fold difference at 15 µM RA was more variable and did not reach statistical significance (mean difference = 2.091 log_2_ units; 95% bootstrap CI: −0.744 to 5.162; *p* = 0.354; *q* = 0.605).

Sensitivity analyses showed complete directional separation between the 5 and 10 µM RA flask-level values and the DMSO values (Supplementary Table S3), yielding Cliff’s *δ* = 1.00 for both comparisons. Median log_2_ differences were 3.163 and 2.649 at 5 and 10 µM RA, respectively, and leave-one-out estimates remained positive. These analyses characterize within-batch consistency but do not overcome the limited batch-level independence of the three flasks per condition.

Given the group size of three flasks versus three flasks, the smallest attainable two-sided exact permutation *p*-value was 0.10. Accordingly, the exact permutation *p*-value was 0.10 for both the 5 and 10 µM YAP1 comparisons despite complete directional separation. The Welch results were therefore not interpreted in isolation; statistical evidence was considered together with the exact-test constraint, effect sizes, bootstrap intervals, sensitivity analyses, and the limited number of flask-level observations.

*YAP1* expression was markedly lower in the DMSO group than in the untreated control, with an estimated fold change of 0.043 (mean difference = −4.525 log_2_ units; Welch *p* = 0.0418); however, this vehicle-associated difference did not remain significant after correction within the four-target vehicle-effect family (*q* = 0.167; Table 3). RA-treated groups remained numerically below the untreated-control level: RA 5 µM versus control, log_2_ difference = −1.243 (fold change = 0.423; Welch *p* = 0.391; *q* = 0.391); RA 10 µM versus control, −1.501 (fold change = 0.353; *p* = 0.313; *q* = 0.391); and RA 15 µM versus control, −2.434 (fold change = 0.185; *p* = 0.316; *q* = 0.391). Thus, the higher *YAP1* values at 5 and 10 µM RA relative to DMSO did not represent expression above the untreated-control level.

No RA-versus-DMSO comparison for *WWTR1* or *NPTN* reached statistical significance after multiplicity correction (all *q* ≥ 0.605 for *WWTR1* and *q* ≥ 0.778 for *NPTN*; Table 3). Their bootstrap intervals included zero, and the leave-one-out estimates were generally unstable or crossed the null value (Supplementary Table S3).

For miR-148a-3p, 5 µM RA produced a large mean difference relative to DMSO, but variation among the three flask-level measurements was substantial and the comparison was not statistically significant (Welch *p* = 0.147; *q* = 0.587; exact permutation *p* = 0.100). The 10 and 15 µM comparisons were also nonsignificant (*q* = 0.605 for both). Because retrospective analysis of the endogenous reference revealed condition-dependent variation in miR-26b-5p Cq values (Section 3.4; Supplementary Table S2), these miR-148a-3p estimates should be regarded as exploratory and normalization-sensitive rather than evidence of a robust RA-associated miRNA response.

### 3.4. Assessment of Endogenous Reference Cq Distributions

Raw Cq values for *ACTB* and miR-26b-5p were examined across all experimental groups (Supplementary Table S2). *ACTB* was evaluated separately within the *YAP1*, *WWTR1*, *and NPTN* qPCR datasets because these measurements originated from target-specific runs. No statistically significant overall treatment-group effect was detected for *ACTB* in the *YAP1*-associated dataset (Welch *p* = 0.123; Kruskal–Wallis *p* = 0.291), the *WWTR1*-associated dataset (Welch *p* = 0.201; Kruskal–Wallis *p* = 0.095), or the *NPTN*-associated dataset (Welch *p* = 0.835; Kruskal–Wallis *p* = 0.815). In contrast, miR-26b-5p Cq values showed a marked condition-associated distribution by Welch’s analysis (*p* = 0.000818), with the DMSO group displaying higher Cq values (mean 28.434 ± 0.433) than the untreated control (24.417 ± 1.840) and the RA groups (23.633 ± 1.421, 23.729 ± 0.334, and 24.718 ± 1.264 at 5, 10, and 15 µM, respectively). The corresponding Kruskal–Wallis test was not significant (*p* = 0.102), consistent with the limited rank-test resolution at *n* = 3 per group. These retrospective analyses do not constitute formal multi-reference-gene validation. They support the absence of a detectable group-level *ACTB* shift within the available run-specific datasets but do not establish stability of miR-26b-5p across conditions.

### 3.5. Assessment of Concentration-Dependent Expression Patterns

The concentration profiles did not provide evidence of a significant monotonic dose–response relationship for any target (Supplementary Table S4 and Figure S1). For *YAP1*, the linear slope was 0.601 −ΔCq units per 5 µM RA increment (HC3-adjusted *p* = 0.397; *q* = 0.793). The quadratic component suggested a possible non-monotonic profile, characterized by stronger responses at 5 and 10 µM and a weaker, more variable response at 15 µM, but this component was not statistically significant after correction (*p* = 0.098; *q* = 0.391).

No significant linear or quadratic concentration trend was detected for *WWTR1*, *NPTN*, or miR-148a-3p (all adjusted *q* ≥ 0.469). Accordingly, the *YAP1* results are more appropriately described as a response occurring within the 5–10 µM concentration range than as evidence of a conventional linear dose-dependent effect.

### 3.6. Cross-Target Relationships and Multivariate Expression Structure

No statistically significant cross-target correlations were detected across the 15 flask-level measurements (Supplementary Table S5). Spearman correlation coefficients ranged from −0.296 for *YAP1–NPTN* to 0.343 for *WWTR1*–miR-148a-3p, and all associations remained nonsignificant after BH-FDR correction (*q* ≥ 0.650). In particular, *YAP1* expression was not significantly correlated with *WWTR1* (*ρ* = −0.236, *p* = 0.398), *NPTN* (*ρ* = −0.296, *p* = 0.283), or miR-148a-3p (*ρ* = 0.179, *p* = 0.524). These exploratory correlations reflect within-batch covariation among 15 flask-level measurements and should be interpreted in the context of the single-batch experimental design.

The standardized-expression heatmap further illustrated the absence of a uniformly coordinated four-target response and highlighted culture-to-culture heterogeneity, particularly for *WWTR1*, *NPTN*, and miR-148a-3p (Supplementary Figure S2). Exploratory principal component analysis produced a first principal component explaining 42.6% of the total variance and a second component explaining 31.0%, together accounting for 73.6% of the four-target expression variation. Nevertheless, the experimental groups did not form clearly separated clusters in the two-dimensional PCA space (Supplementary Figure S3).

### 3.7. Integrated Summary of the Experimental Findings

Across the four-target panel, the only design-defined RA-versus-DMSO comparisons meeting the BH-FDR threshold were the higher *YAP1* transcript levels at 5 and 10 µM RA. However, *YAP1* was markedly lower in the DMSO group than in untreated cultures, and none of the RA groups differed significantly from the untreated control. The overall pattern is therefore compatible with a selective *YAP1* response relative to the DMSO vehicle background, including the possibility of partial attenuation of a vehicle-associated reduction, rather than simple induction above untreated baseline. No statistically robust coordinated or monotonic response was detected across *WWTR1*, *NPTN*, and miR-148a-3p; interpretation of the miR-148a-3p estimates is further constrained by condition-dependent variation in miR-26b-5p.

## 4. Discussion

The principal finding was a selective *YAP1* transcriptional difference relative to the DMSO vehicle background after seven days of RA exposure. *YAP1* levels were 9.73-fold and 8.14-fold higher at 5 and 10 µM RA, respectively, than in DMSO, and both Welch comparisons remained significant after BH-FDR correction. However, the DMSO group itself showed markedly lower *YAP1* expression than the untreated control (0.043-fold), and that vehicle-associated contrast did not remain significant after correction. Importantly, direct comparisons with the untreated control showed that none of the RA-treated groups differed significantly from the untreated control and that their mean *YAP1* levels remained below untreated baseline. The data should therefore not be described as simple RA-induced upregulation above baseline. A plausible alternative interpretation is that RA at 5–10 µM partially attenuated or reversed a DMSO-associated reduction in YAP1. Because the experiment used a fixed 0.1% DMSO concentration in all RA groups and did not include lower vehicle concentrations, the relative contributions of RA, vehicle background, and their interaction cannot be resolved definitively.

The seven-day time point captures an early transcriptional phase of RA exposure rather than a defined developmental state. Classical NTERA-2 studies generated mature neuronal populations using substantially longer, multistage differentiation procedures [9,10], whereas the present cultures were examined without neuronal enrichment. Hindley et al. associated YAP with proliferative and less differentiated neural populations and showed that YAP manipulation can influence neural cell fate [17]. Nevertheless, the present study did not measure pluripotency, neural crest, neural progenitor, or neuronal lineage markers. Consequently, *YAP1* cannot be designated here as a marker of a specific ‘early RA-responsive state’; the supported conclusion is limited to an RA-associated *YAP1* transcriptional response relative to the vehicle background, without assignment of lineage identity.

The concentration profile was non-monotonic. Differences relative to DMSO were strongest at 5 and 10 µM, whereas the 15 µM group showed greater flask-to-flask dispersion, and neither the linear nor quadratic concentration trend was significant after correction. Thus, the current data do not support a conventional dose–response relationship. Because RA responses depend on developmental stage, receptor context, exposure duration, and pathway crosstalk [1,2,3,4,5,6,7], a non-monotonic pattern is biologically plausible, but the present single-time-point, single-batch design cannot distinguish a true concentration window from experimental variability.

The absence of a corresponding *WWTR1* change argues against treating YAP and TAZ as interchangeable transcriptional readouts. Both proteins integrate Hippo and mechanical inputs [12,13], yet *WWTR1* transcript levels were highly variable and none of its RA-versus-DMSO comparisons survived multiplicity correction. Importantly, *YAP1* mRNA abundance alone is not evidence of Hippo-pathway activation. Pathway output depends on YAP/TAZ protein abundance, phosphorylation, stability, subcellular localization, and TEAD-dependent transcription [12,13], none of which was measured here. Accordingly, the present findings are described as a selective *YAP1* transcript-level response rather than evidence of Hippo-pathway activation.

The *NPTN* result provides a useful contrast with a different human neural differentiation system. Desrivières et al. observed an approximately 1.5-fold increase in *NPTN* at differentiation day 7 in human neural progenitor cells, at a stage when neurites were well developed and adhesion -and synaptic-transmission-related genes were being induced [21]. Neuroplastin’s established participation in PMCA-dependent Ca^2+^ handling and glutamatergic synaptic plasticity further places it within functional neuronal organization rather than defining it as an obligatory immediate RA-response gene [19,20]. In the present NTERA-2 cultures, *NPTN* remained broadly similar to DMSO at all three RA concentrations. This difference is not necessarily inconsistent: the studies used different cell models, media and differentiation protocols, and the present cultures were not shown quantitatively to have reached a neurite-rich stage. Rather, the lack of *NPTN* induction indicates that the *YAP1* response was not accompanied by this particular neuroplastin-associated transcriptional feature. 

The miR-148a-3p findings require particular caution. None of the RA-versus-DMSO comparisons was statistically significant after correction, and the 5 µM group showed marked within-group heterogeneity. In addition, retrospective examination of the reference assay revealed a condition-associated shift in miR-26b-5p Cq values, especially in the DMSO group. Although miR-26b-5p has previously been evaluated as a candidate endogenous control in miRNA RT-qPCR studies [29], reference suitability is context-dependent [28,29], and miR-26b-5p itself has a documented role in neuronal differentiation [30]. Accordingly, stability of miR-26b-5p cannot be assumed under the present RA-exposure conditions, and the normalized miR-148a-3p results should be regarded as exploratory. A future validation should use multiple candidate reference miRNAs and a formal stability algorithm before drawing mechanistic conclusions from miRNA normalization.

Culture morphology and density are additional potential confounders. The representative fields showed qualitative differences in apparent density, intercellular spacing, and spreading, while MTT-based metabolic viability remained at 95–97% of the untreated control reference. MTT was not a proliferation assay, and proliferation rate was not independently measured. This distinction is important because YAP/TAZ activity is sensitive to cell density, cell–cell contact, cytoskeletal tension, and mechanical context [12,17]. The relatively low starting density compared with routine NTERA-2 maintenance recommendations may also have influenced the culture state. Because confluence, cell count, cell area, neurite morphology, and proliferation were not quantified, the present study cannot separate RA-associated transcriptional effects from density- or mechanics-associated changes.

The study has several strengths, including a matched DMSO vehicle condition, transparent reporting of individual flask-level measurements, design-defined vehicle-referenced contrasts, and multiple sensitivity analyses. However, the three flasks per condition originated from the same passage and common starting suspension and therefore represent parallel within-batch cultures rather than independent experimental batches initiated from separate passages, cell preparations, or experimental days. This constrained batch-level independence, together with the small group size, limits precision and raises a pseudoreplication concern. Additional limitations include the single cell line and time point, focused transcript panel, absence of quantitative morphometry and proliferation measurements, lack of YAP/TAZ protein-level and functional pathway assays, and lack of formal multi-reference validation. The raw-Cq analysis did not detect a statistically significant treatment-group shift in *ACTB* within the available target-specific runs, whereas condition-dependent variation was identified for miR-26b-5p, specifically limiting interpretation of the normalized miR-148a-3p findings. These limitations substantially restrict mechanistic inference and the generalizability of the findings.

Future validation should prioritize experiments initiated from independent experimental batches using separate cell passages or starting cell preparations, include lower DMSO vehicle concentrations or alternative vehicle conditions, and use density-matched cultures with quantitative assessment of confluence, cell number, cell area, and proliferation. A time-resolved design incorporating pluripotency, neural crest, neural progenitor, and neuronal markers would be required to assign cell-state identity. Parallel measurement of YAP/TAZ protein abundance, phosphorylation, subcellular localization, and TEAD-responsive genes would determine whether the transcript-level *YAP1* pattern translates into altered Hippo-pathway output. For miRNA analyses, multiple candidate endogenous controls should be prospectively validated across treatment conditions.

## 5. Conclusions

In conclusion, seven-day RA exposure was associated with higher *YAP1* transcript levels at 5 and 10 µM relative to the 0.1% DMSO vehicle background, without a significant monotonic dose–response and without corresponding statistically robust changes in *WWTR1* or *NPTN*. Because *YAP1* expression was markedly lower in the DMSO group than in untreated cultures and none of the RA groups differed significantly from untreated control, the pattern is also compatible with partial attenuation of a vehicle-associated *YAP1* reduction rather than induction above baseline. The study does not establish a defined neural developmental state or Hippo-pathway activation, and its interpretation is limited by within-batch replication, small group size, unquantified density/morphology and proliferation, absence of protein-level validation, and condition-dependent variation in the miR-26b-5p normalizer. The findings therefore define a focused, hypothesis-generating YAP1 transcriptional response that requires validation in independent experimental batches and with orthogonal molecular readouts.

## Figures and Tables

**Table 1:** Descriptive statistics for normalized target expression across experimental conditions.

Target	Group	Mean −ΔCq ± SD	Median	95% CI for Mean	Fold Change vs. DMSO
*YAP1*	Control	0.565 ± 1.948	1.668	−4.274 to 5.403	23.02
*YAP1*	DMSO	−3.960 ± 0.786	−3.607	−5.912 to −2.009	1.00
*YAP1*	RA 5 µM	−0.678 ± 0.831	−0.444	−2.741 to 1.385	9.73
*YAP1*	RA 10 µM	−0.936 ± 0.600	−0.958	−2.428 to 0.555	8.14
*YAP1*	RA 15 µM	−1.870 ± 3.024	−2.106	−9.382 to 5.643	4.26
*WWTR1*	Control	−1.992 ± 4.125	−0.334	−12.240 to 8.256	0.004
*WWTR1*	DMSO	5.856 ± 4.593	4.976	−5.554 to 17.267	1.00
*WWTR1*	RA 5 µM	7.925 ± 4.036	9.734	−2.101 to 17.951	4.19
*WWTR1*	RA 10 µM	1.906 ± 4.077	−0.404	−8.221 to 12.033	0.065
*WWTR1*	RA 15 µM	1.235 ± 4.073	2.134	−8.883 to 11.352	0.041
*NPTN*	Control	0.741 ± 0.329	0.782	−0.077 to 1.559	0.60
*NPTN*	DMSO	1.481 ± 1.712	0.947	−2.772 to 5.734	1.00
*NPTN*	RA 5 µM	0.558 ± 3.846	0.848	−8.997 to 10.112	0.53
*NPTN*	RA 10 µM	1.464 ± 5.683	4.547	−12.654 to 15.581	0.99
*NPTN*	RA 15 µM	0.900 ± 1.053	1.238	−1.716 to 3.517	0.67
miR-148a-3p	Control	−11.381 ± 3.753	−10.178	−20.704 to −2.058	1.14
miR-148a-3p	DMSO	−11.574 ± 0.907	−11.051	−13.828 to −9.320	1.00
miR-148a-3p	RA 5 µM	6.509 ± 13.565	13.327	−27.187 to 40.206	277,823
miR-148a-3p	RA 10 µM	−9.271 ± 3.786	−9.263	−18.675 to 0.134	4.94
miR-148a-3p	RA 15 µM	−7.199 ± 6.778	−3.292	−24.036 to 9.638	20.76

Data are presented as mean −ΔCq ± SD, median, and 95% CI (three parallel flask-level measurements per group from a single experimental batch). Fold changes were calculated relative to the DMSO vehicle control. Higher −ΔCq values indicate greater normalized expression. RA, retinoic acid; DMSO, dimethyl sulfoxide.

**Table 2:** Overall comparisons of normalized expression among experimental groups.

Target	Welch *F*	Welch *p*	Welch *q*	Permutation *p*	Permutation *q*	Kruskal–Wallis *p*
*YAP1*	6.742	0.0324	0.1295	0.0857	0.1341	0.1241
*WWTR1*	1.927	0.2445	0.4889	0.1006	0.1341	0.1450
*NPTN*	0.108	0.9735	0.9735	0.9972	0.9972	0.9197
miR-148a-3p	1.258	0.4095	0.5460	0.0609	0.1341	0.2457

The *q* values were calculated using the Benjamini–Hochberg false-discovery rate correction. Statistical significance was defined as *q* < 0.05.

**Table 3:** Pairwise comparisons of normalized target expression.

Target	Comparison	Log_2_ Difference	Fold Change	Bootstrap 95% CI	Hedges’ *g*	Welch *p*	FDR *q*	Exact *p*
*YAP1*	DMSO vs. control	−4.525	0.043	−6.139 to −2.340	−2.44	0.0418	0.1671	0.10
*YAP1*	RA 5 vs. DMSO	3.282	9.73	2.263 to 4.301	3.25	0.0077	**0.0462**	0.10
*YAP1*	RA 10 vs. DMSO	3.024	8.14	2.142 to 3.924	3.46	0.0073	**0.0462**	0.10
*YAP1*	RA 15 vs. DMSO	2.091	4.26	−0.744 to 5.162	0.76	0.3539	0.6054	0.30
*WWTR1*	DMSO vs. control	7.848	230.42	2.250 to 13.446	1.44	0.0933	0.1865	0.10
*WWTR1*	RA 5 vs. DMSO	2.069	4.19	−3.431 to 7.568	0.38	0.5899	0.7776	0.70
*WWTR1*	RA 10 vs. DMSO	−3.951	0.065	−9.338 to 1.437	−0.73	0.3285	0.6054	0.30
*WWTR1*	RA 15 vs. DMSO	−4.622	0.041	−10.307 to 1.063	−0.85	0.2631	0.6054	0.40
*NPTN*	DMSO vs. control	0.740	1.67	−0.641 to 2.568	0.48	0.5339	0.7119	0.80
*NPTN*	RA 5 vs. DMSO	−0.924	0.53	−4.907 to 2.769	−0.25	0.7313	0.7977	0.80
*NPTN*	RA 10 vs. DMSO	−0.017	0.99	−6.293 to 4.426	−0.003	0.9964	0.9964	1.00
*NPTN*	RA 15 vs. DMSO	−0.581	0.67	−2.496 to 1.193	−0.33	0.6480	0.7776	0.70
miR-148a-3p	DMSO vs. control	−0.193	0.87	−3.197 to 4.013	−0.06	0.9380	0.9380	1.00
miR-148a-3p	RA 5 vs. DMSO	18.084	2.78 × 10^5^	2.463 to 26.887	1.50	0.1466	0.5865	0.10
miR-148a-3p	RA 10 vs. DMSO	2.304	4.94	−1.486 to 6.085	0.67	0.4036	0.6054	0.50
miR-148a-3p	RA 15 vs. DMSO	4.376	20.76	−3.450 to 8.815	0.72	0.3798	0.6054	0.40
*YAP1*	RA 5 vs. control	−1.243	0.423	−2.912 to 0.855	−0.66	0.3915	0.3915	0.40
*YAP1*	RA 10 vs. control	−1.501	0.353	−3.033 to 0.559	−0.83	0.3126	0.3915	0.40
*YAP1*	RA 15 vs. control	−2.434	0.185	−5.578 to 0.709	−0.77	0.3164	0.3915	0.20

Log_2_ differences were calculated on the −ΔCq scale and converted to fold change as 2^(log^_2_^difference)^. BH-FDR was applied within the analytical families defined in the Statistical Analysis section; *YAP1* RA-versus-untreated comparisons constituted a separate three-comparison family. Exact *p*-values are from two-sided permutation tests. The extreme miR-148a-3p estimate at 5 µM RA should be interpreted cautiously because of small sample size, high variability, and condition-dependent miR-26b-5p variation; it was not significant after FDR correction. Bootstrap 95% CIs are descriptive.

## Data Availability

The data supporting the findings of this study are available from the corresponding author upon reasonable request. Processed summary data are provided within the article and Supplementary Materials.

## Supplementary Materials

The following supporting information can be downloaded at: https://www.mdpi.com/article/10.3390/life16101668/s1, Supplementary Table S1. Primer and probe sequences used for RT-qPCR analysis. Supplementary Table S2. Raw Cq distributions and group-level assessment of the endogenous reference assays used for RT-qPCR normalization. Supplementary Table S3. Robustness analyses of retinoic acid effects relative to the DMSO control. Supplementary Table S4. Retinoic acid dose-trend analysis. Supplementary Table S5. Exploratory correlations between normalized target-expression levels. Supplementary Figure S1. Expression profiles of YAP1, WWTR1, NPTN, and miR-148a-3p across retinoic acid concentrations. Supplementary Figure S2. Heatmap of normalized expression profiles across individual flask-level NTERA-2 samples. Supplementary Figure S3. Principal component analysis (PCA) of the four-target expression profile.

## Abbreviations

The following abbreviations are used in this manuscript:

*ACTB*	*Actin beta*
BH-FDR	Benjamini–Hochberg false discovery rate
cDNA	Complementary DNA
CI	Confidence interval
Cq	Quantification cycle
DMSO	Dimethyl sulfoxide
DMEM	Dulbecco’s Modified Eagle Medium
miRNA	MicroRNA
MTT	3-(4,5-dimethylthiazol-2-yl)-2,5-diphenyltetrazolium bromide
*NPTN*	*Neuroplastin*
PCA	Principal component analysis
PBS	Phosphate-buffered saline
PMCA	Plasma membrane Ca^2+^-ATPase
RA	Retinoic acid
RAR	Retinoic acid receptor
RT-qPCR	Reverse transcription quantitative polymerase chain reaction
SD	Standard deviation
TAZ	Transcriptional coactivator with PDZ-binding motif
TEAD	TEA domain transcription factor
*WWTR1*	*WW domain-containing transcription regulator 1*
*YAP1*	*Yes-associated protein 1*
