# Supplementary material for: Selective YAP1 Transcriptional Response to Graded Retinoic Acid Exposure in NTERA-2 Cells
Source: Life (Basel). 2026 Oct 6;16(10):1668. doi: 10.3390/life16101668 (PMC13705999; doi:10.3390/life16101668)
Supplement: Supplementary file 1 [file life-16-01668-s001.zip › life-4566188 Supplementary Tables.pdf]

Supplementary Tables

Supplementary Table S1. Primer and probe sequences used for RT-qPCR analysis.

Gene(NCBI Gene ID)	Primers and probe sequences
YAP1 (10413)	F: 5′-CGTCCAGCAAGATACTTTAATCCTCTAT-3′<br>R: 5′-ACTGTGAAAGAGGTCAGCAATACATT-3′<br>PR: 5′-FAM-TTGCTCTTCCTTGTCCATTGCTGCTGT-BHQ-1-3′
WWTR1 (25937)	F: 5′-GCCTTAGCCTCCCGAGTAGCT-3′<br>R: 5′-AAACCCCGTCTCTACTCAAAATACA-3′<br>PR: 5′-FAM-CACCTGCCACCACGTCCGGCTA-BHQ-1-3′
NPTN (27020)	F: 5′-CCAGCTCTCACACCCTTACA-3′<br>R: 5′-ACTCCGTTATGGCCAGTGA-3′<br>PR: 5′-FAM-CCTAAGCAAACGCCACCAT-BHQ-1-3′
miR-148a-3p (406940)	F: 5′-CCGCTCAGTGCACTACAGA-3′<br>R: 5′-GTGCAGGGTCCGAGGTAT-3′<br>PR: 5′-6-FAM-TG(pdC)ATA(pdC)GA(pdC)(pdC)AAAGT-ZNA-4-BHQ-1-3′
ACTB (60)	F: 5′-GGCACCCAGCACAATGAAG-3′<br>R: 5′-GCCGATCCACACGGAGTACT-3′<br>PR: 5′-FAM-TCAAGATCATTGCTCCTCCTGAGCGC-BHQ-1-3′
miR-26b-5p (407017)	F: 5′-GCCGCTTCAAGTAATTCAGG-3’<br>R: 5′-GTCGTATGCAGTGCAGGGTCCGAGGTATTCGCACTGCATACGACACCTAT-3′<br>PR: 5′-FAM-TG(pdC)ATA(pdC)GA(pdC)A(pdC)CTATCC-ZNA-4-BHQ-1-3′

F, forward primer; R, reverse primer; PR, probe; FAM/6-FAM, 6-carboxyfluorescein; BHQ-1, Black Hole Quencher 1; pdC, C-5-propynyl-deoxycytidine; ZNA-4, four cationic spermine units incorporated into a zip nucleic acid probe. NCBI Gene IDs were obtained from the NCBI Gene database (https://www.ncbi.nlm.nih.gov/gene/; accessed March 2026).

Supplementary Table S2. Raw Cq distributions and group-level assessment of the endogenous reference assays used for RT-qPCR normalization.

Panel A. Raw endogenous-reference Cq values.

Experimental group	Flask	*ACTB* Cq – *YAP1* run	*ACTB* Cq – *WWTR1* run	*ACTB* Cq – *NPTN* run	miR-26b-5p Cq – miR-148a-3p run
DMSO	1	32.523	38.711	33.465	28.933
DMSO	2	29.801	34.568	34.485	28.199
DMSO	3	30.761	37.540	39.071	28.169
Control	1	36.217	35.282	35.355	26.249
Control	2	34.128	32.051	36.895	24.434
Control	3	31.519	33.756	36.899	22.569
RA 5 µM	1	28.764	35.260	25.474	23.069
RA 5 µM	2	27.770	33.258	29.580	25.249
RA 5 µM	3	35.712	33.415	39.168	22.580
RA 10 µM	1	33.896	36.279	32.835	23.921
RA 10 µM	2	35.860	35.357	40.403	23.923
RA 10 µM	3	35.295	36.777	38.874	23.344
RA 15 µM	1	35.848	35.645	35.785	26.016
RA 15 µM	2	30.525	36.012	34.884	23.490
RA 15 µM	3	30.476	35.254	37.214	24.649

Supplementary Table S2. Raw Cq distributions and group-level assessment of the endogenous reference assays used for RT-qPCR normalization.

Panel B. Group-level descriptive and overall comparisons.

**Reference dataset**	**Group**	**Mean Cq**	**SD**	**Median**	**Min**	**Max**	**Welch ANOVA *p***	**Kruskal–Wallis *p***
ACTB – YAP1 run	Control	33.955	2.354	34.128	31.519	36.217	0.123	0.291
ACTB – YAP1 run	DMSO	31.028	1.381	30.761	29.801	32.523	0.123	0.291
ACTB – YAP1 run	RA 5 µM	30.749	4.327	28.764	27.770	35.712	0.123	0.291
ACTB – YAP1 run	RA 10 µM	35.017	1.011	35.295	33.896	35.860	0.123	0.291
ACTB – YAP1 run	RA 15 µM	32.283	3.087	30.525	30.476	35.848	0.123	0.291
ACTB – WWTR1 run	Control	33.696	1.616	33.756	32.051	35.282	0.201	0.095
ACTB – WWTR1 run	DMSO	36.940	2.136	37.540	34.568	38.711	0.201	0.095
ACTB – WWTR1 run	RA 5 µM	33.978	1.113	33.415	33.258	35.260	0.201	0.095
ACTB – WWTR1 run	RA 10 µM	36.138	0.720	36.279	35.357	36.777	0.201	0.095
ACTB – WWTR1 run	RA 15 µM	35.637	0.379	35.645	35.254	36.012	0.201	0.095
ACTB – NPTN run	Control	36.383	0.890	36.895	35.355	36.899	0.835	0.815
ACTB – NPTN run	DMSO	35.674	2.986	34.485	33.465	39.071	0.835	0.815
ACTB – NPTN run	RA 5 µM	31.407	7.028	29.580	25.474	39.168	0.835	0.815
ACTB – NPTN run	RA 10 µM	37.371	4.002	38.874	32.835	40.403	0.835	0.815
ACTB – NPTN run	RA 15 µM	35.961	1.175	35.785	34.884	37.214	0.835	0.815
miR-26b-5p	Control	24.417	1.840	24.434	22.569	26.249	0.000818	0.102
miR-26b-5p	DMSO	28.434	0.433	28.199	28.169	28.933	0.000818	0.102
miR-26b-5p	RA 5 µM	23.633	1.421	23.069	22.580	25.249	0.000818	0.102
miR-26b-5p	RA 10 µM	23.729	0.334	23.921	23.344	23.923	0.000818	0.102
miR-26b-5p	RA 15 µM	24.718	1.264	24.649	23.490	26.016	0.000818	0.102

ACTB measurements originated from the corresponding target-specific qPCR runs and were evaluated separately rather than pooled across runs. No statistically significant overall treatment-group effect was detected for *ACTB* within the *YAP1-, WWTR1-, or NPTN*-associated datasets. In contrast, miR-26b-5p Cq values showed a condition-associated distribution, driven predominantly by higher Cq values in the DMSO group. These analyses represent a retrospective assessment of raw reference-assay Cq distributions and should not be interpreted as formal multi-reference-gene validation. Three parallel flask cultures were analysed per condition; all originated from the same passage and common starting cell suspension. Welch ANOVA and Kruskal–Wallis *p* values represent overall comparisons across the five experimental groups within each reference dataset.

Supplementary Table S3. Robustness analyses of retinoic acid effects relative to the DMSO control.

**Target**	**Comparison**	**Median $\log_2$ difference**	**Cliff's δ**	**Leave-one-out $\log_2$ difference range**
YAP1	RA 5 µM vs DMSO	3.163	1.000	2.49 to 4.02
YAP1	RA 10 µM vs DMSO	2.649	1.000	2.27 to 3.59
YAP1	RA 15 µM vs DMSO	1.501	0.556	0.07 to 3.81
WWTR1	RA 5 µM vs DMSO	4.758	0.111	−1.38 to 6.87
WWTR1	RA 10 µM vs DMSO	−5.380	−0.556	−8.35 to −0.27
WWTR1	RA 15 µM vs DMSO	−2.842	−0.556	−8.44 to 0.09
NPTN	RA 5 µM vs DMSO	−0.099	−0.111	−3.46 to 2.03
NPTN	RA 10 µM vs DMSO	3.600	0.333	−2.45 to 4.22
NPTN	RA 15 µM vs DMSO	0.291	−0.111	−1.69 to 0.97
miR-148a-3p	RA 5 µM vs DMSO	24.378	1.000	13.16 to 26.16
miR-148a-3p	RA 10 µM vs DMSO	1.788	0.333	−0.11 to 4.46
miR-148a-3p	RA 15 µM vs DMSO	7.759	0.333	1.89 to 8.55

Differences were calculated on the −ΔCq scale relative to the DMSO control. Positive values and positive Cliff's δ estimates indicate higher expression in the RA group. Leave-one-out ranges were obtained after sequential exclusion of individual observations.

Supplementary Table S4. Retinoic acid dose-trend analysis.

**Target**	**Linear $\log_2$ slope per 5 µM**	**Fold change per 5 µM**	**Linear *p***	**Linear *q***	**Quadratic *p***	**Quadratic *q***
YAP1	0.601	1.52	0.3966	0.7933	0.0978	0.3914
WWTR1	−1.988	0.252	0.1360	0.5439	0.6477	0.8636
NPTN	−0.084	0.944	0.8759	0.9086	0.9340	0.9340
miR-148a-3p	−0.265	0.832	0.9086	0.9086	0.2347	0.4694

DMSO was coded as 0 µM RA. Linear and quadratic trends were assessed using regression models with HC3 robust standard errors. The *q* values were calculated using the Benjamini–Hochberg correction; $q < 0.05$ was considered statistically significant.

Supplementary Table S5. Exploratory correlations between normalized target-expression levels.

**Target pair**	**Spearman ρ**	***p***	**BH-FDR *q***
YAP1–WWTR1	−0.236	0.3977	0.6495
YAP1–NPTN	−0.296	0.2834	0.6495
YAP1–miR-148a-3p	0.179	0.5243	0.6495
WWTR1–NPTN	−0.064	0.8199	0.8199
WWTR1–miR-148a-3p	0.343	0.2109	0.6495
NPTN–miR-148a-3p	−0.171	0.5413	0.6495

Correlations were evaluated using Spearman's rank correlation across all flask-level measurements (n = 15) from the single experimental batch. The *q* values were calculated using the Benjamini–Hochberg false-discovery rate correction.
