# Supplementary material for: Selective YAP1 Transcriptional Response to Graded Retinoic Acid Exposure in NTERA-2 Cells
Source: Life (Basel). 2026 Oct 6;16(10):1668. doi: 10.3390/life16101668 (PMC13705999; doi:10.3390/life16101668)
Supplement: Supplementary file 1 [file life-16-01668-s001.zip › life-4566188 Supplementary Figures .pdf]

Supplementary Figures

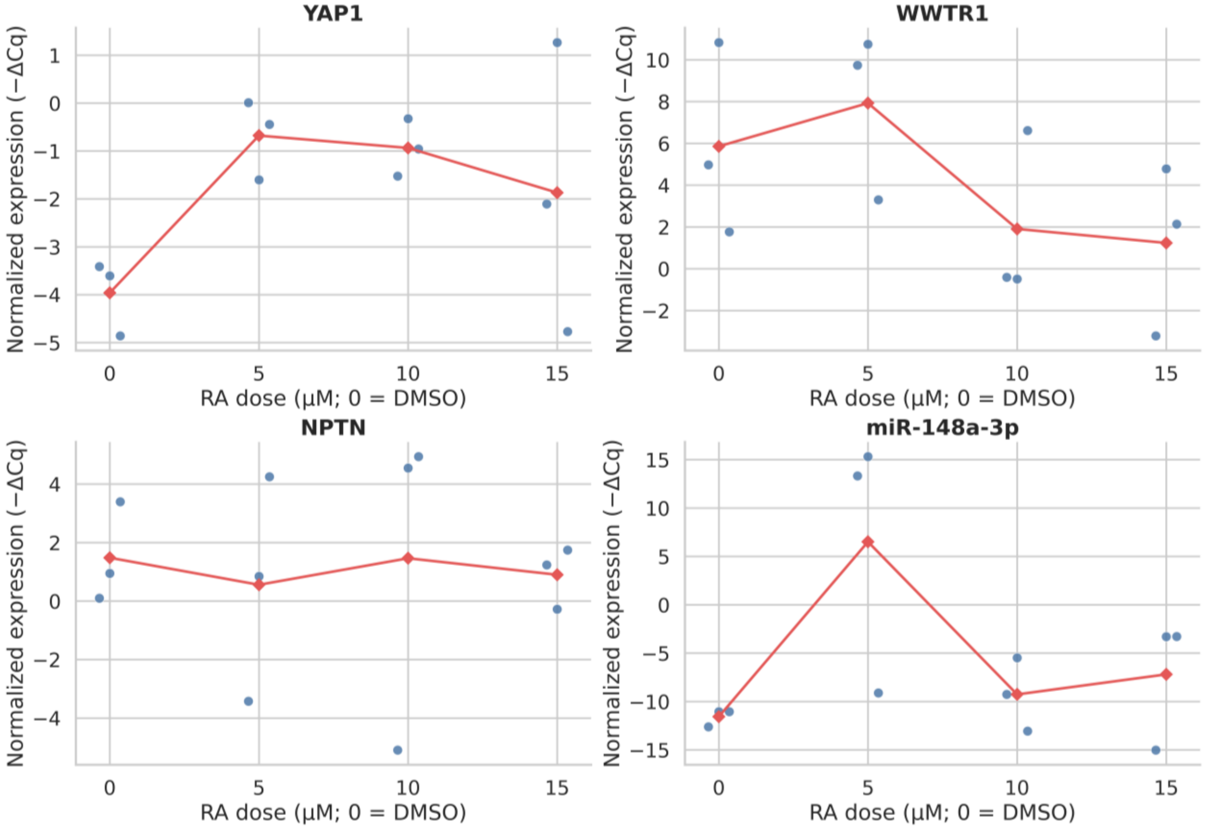

Supplementary Figure S1. Expression profiles of *YAP1, WWTR1, NPTN*, and miR-148a-3p across retinoic acid concentrations. Blue points represent individual flask-level measurements, and red diamonds connected by lines indicate group means. Expression is presented as −ΔCq values, with 0 µM representing the DMSO vehicle control.

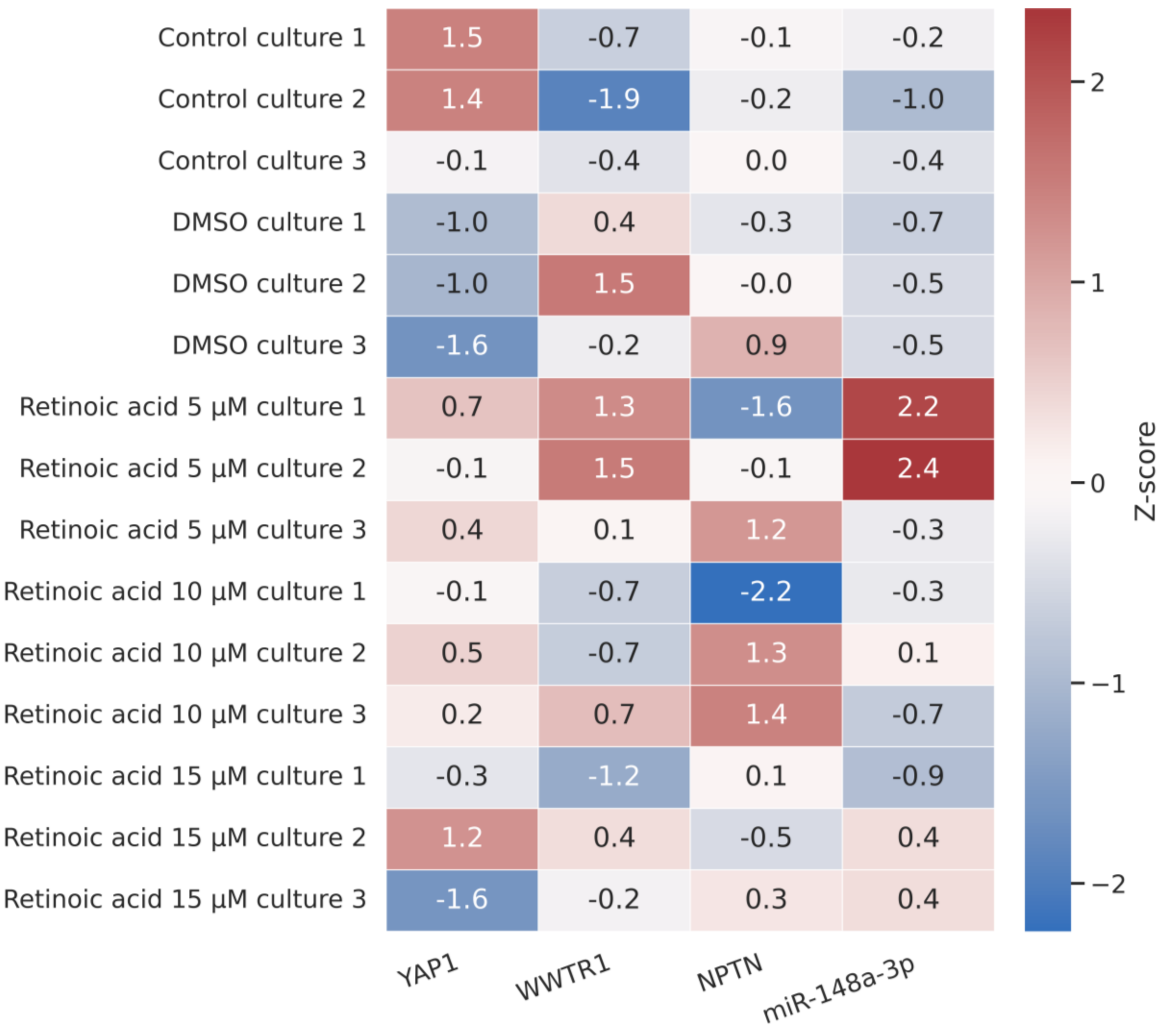

Supplementary Figure S2. Heatmap of normalized expression profiles across individual flask-level NTERA-2 samples. Values represent target-specific Z-scores calculated from −ΔCq measurements. Red indicates expression above and blue indicates expression below the target-specific mean. The labels “culture 1–3” in the heatmap denote the three parallel flask-level samples within each condition from the single experimental batch.

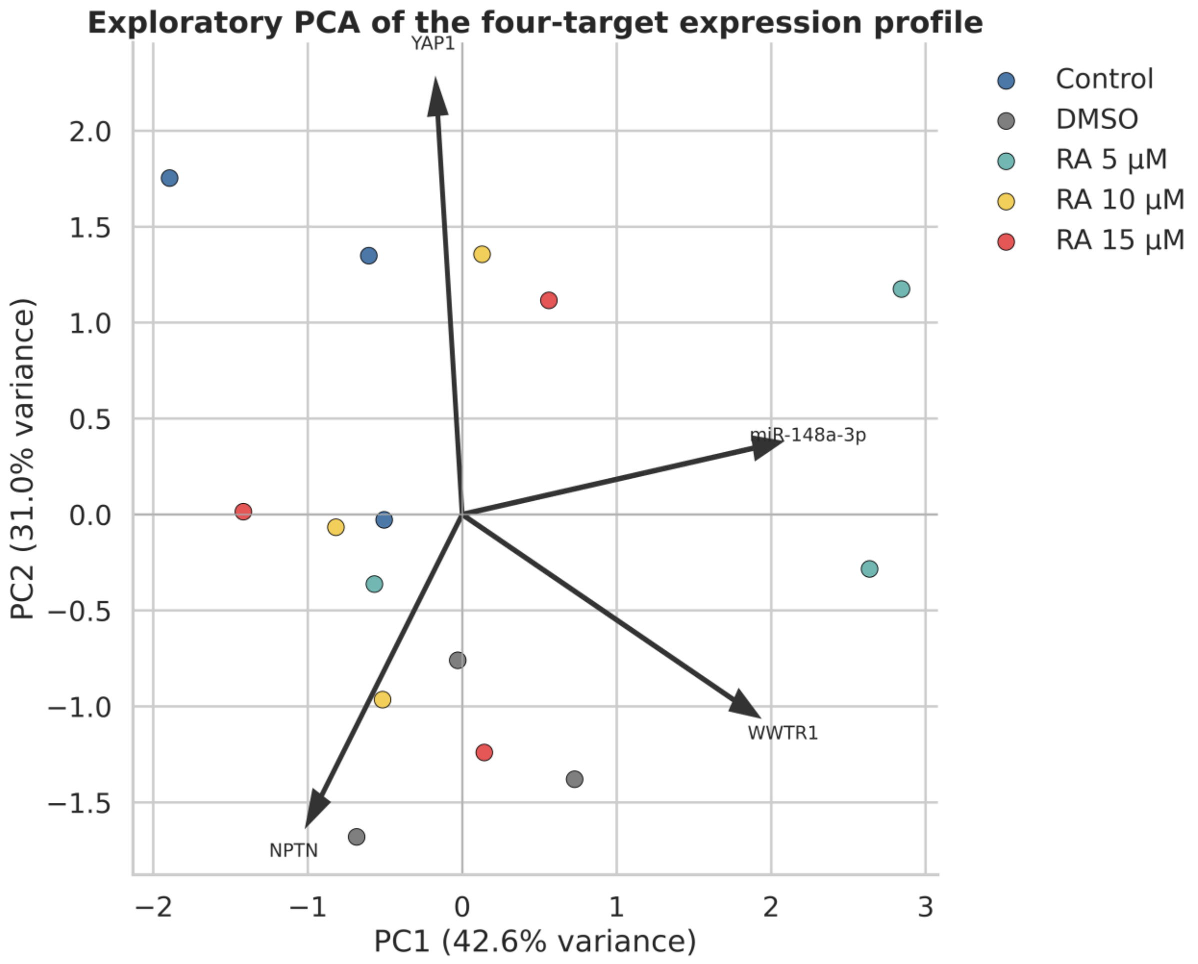

Supplementary Figure S3. Principal component analysis (PCA) of the four-target expression profile. Points represent individual flask-level measurements, coloured by experimental group, and arrows indicate target loadings. PC1 and PC2 explained 42.6% and 31.0% of the total variance, respectively.
